# Unlocking the Therapeutic and Antimicrobial Potential of *Prunus armeniaca* L. Seed Kernel Oil

**DOI:** 10.1155/2024/5589506

**Published:** 2024-11-07

**Authors:** Zeenat Hamid, Ali Akbar, Kashif Kamran, Jahangir Khan Achakzai, Ling Shing Wong, Muhammad Bilal Sadiq

**Affiliations:** ^1^Department of Microbiology, University of Balochistan, Quetta, Pakistan; ^2^Centre for Biotechnology and Microbiology, University of Swat, Charbagh 19120, Khyber Pakhtunkhwa, Pakistan; ^3^Faculty of Health and Life Sciences, INTI International University, Nilai, Negeri Sembilan 11 71800, Malaysia; ^4^Department of Zoology, University of Balochistan, Quetta, Pakistan; ^5^Discipline of Biochemistry, Department of Natural and Basic Sciences, University of Turbat, Kech 92600, Balochistan, Pakistan; ^6^School of Life Sciences, Forman Christian College (A Chartered University), Lahore, Pakistan

**Keywords:** bioactive compounds, food security, harvest losses, health, improved nutrition

## Abstract

The *Prunus armeniaca* L. (bitter apricot) is an apricot fruit tree categorized on the basis of the bitter taste of its seed kernel. In this study, the functional, medicinal, and therapeutic potential of bitter apricot seed kernel oil (BASKO) was evaluated. The qualitative screening of BASKO was performed using standard methodologies. The chemical profile of the oil was analyzed with the help of Fourier transform infrared (FTIR) and gas chromatography and mass spectrometry (GC-MS). Results revealed the presence of different phytochemical constituents comprising steroids, flavonoids, terpenoids, alkaloids, and cardiac glycosides. The antioxidant activity of the oil was determined by a 2,2,diphenyl-1picrylhydrazyl (DPPH) radical inhibition essay. Total phenolic and flavonoid contents were 10.6 ± 1.32 mg GAE/g and 4.75 ± 0.11 mg QE/g, respectively. DPPH inhibition of 89.5% was achieved at 1000 *μ*g/mL of BASKO, with IC_50_ = 90.44 *μ*g/mL (83.47–96.67 *μ*g/mL with 95% CI). The antimicrobial potential of the BASKO revealed the inhibition of *Escherichia coli* (20.3 ± 2.08 mm), *Salmonella typhi* (19.3 ± 2.51 mm), *Klebsiella pneumoniae* (16.6 ± 1.52 mm), *Pseudomonas aeruginosa* (17 ± 2 mm), and *Staphylococcus aureus* (25 ± 1.01 mm). The minimum inhibitory concentration (MIC) value was 250 *μ*L/mL for *K. pneumoniae*, *S. typhi*, *P. aeruginosa*, and *S. aureus*, whereas 62.5 *μ*L/mL for *E. coli*. Moreover, BASKO showed antifungal potential against *Trichophyton tonsurans* (77.3 ± 2.08%), *Epidermophyton floccosum* (69.6 ± 3.51%), *Aspergillus niger* (74.3 ± 2.56%), *Aspergillus flavus* (90 ± 3%), and *Mucor mucedo* (78.3 ± 2.51%). Antileishmanial activity of oil was evaluated against *Leishmania major* by MTT assay, and an IC_50_ value of 89.75 *μ*g/mL was observed. The study revealed that BASKO is a good source of biologically active compounds to be used as functional, therapeutical, and antimicrobial agents in food and pharmaceutical products.

## 1. Introduction

The *Prunus armeniaca*, commonly called apricot, is grown in temperate regions and is among the most famous fruits grown globally, particularly in Western and Central Asia, where Turkey and Uzbekistan dominate world apricot production [1, 2]. Pakistan holds the sixth position in its production, and it is mostly cultivated in different areas of Khyber Pakhtunkhwa and Balochistan provinces [3]. The apricot is categorized into three categories due to its seed taste: sugary apricot, partially bitter apricot, and bitter apricot [4]. The *Rosacea* family and the genus *Prunus* include the wild apricot, also known as the bitter apricot. The mature fruit, which is from the *Prunus armeniaca* tree, is eaten both fresh and dried [5, 6]. Its kernel contains pale yellow oil, about 47%, with a saponification range of 187.3–199 and a refractive index range of 1.464–1.480 [7]. Its oil encompasses numeral therapeutical properties comprising antioxidant, antitumor, antifungal, antiasthmatic, antiviral, antibacterial, antileishmanial, anti-inflammatory, and insecticidal. Moreover, it is utilized in the leather, biodiesel, pharmaceutical, food, bakery, and cosmetics industries. It can be used as functional food because of its own health benefits [8].

A novel avenue for the analysis of food components has been made possible by the protective effects of dietary antioxidants in contrast to human degenerative illnesses [9]. Like bitter apricot seed kernel oil, its shell and leaves also exhibit medicinal attributes such as antioxidant, anticancer, antiasthmatic, antimicrobial, and anti-inflammatory effects [10, 11]. The Japanese apricot is used in a particular drink for the prevention and cure of cancer cell proliferation [12]. Flavonoids, galangin, p-coumaric, chrysin, benzoic acid, pinocembrin, and caffeic acids found in apricot seeds have been proven to have antiviral action against HSV-1 [13]. Different biologically important constituents such as flavonoids, vitamins, stilbene, phenolics, alkaloids, betalains, tannins, amines, and secondary metabolites are responsible for functional activities [14]. The literature testifies that phenolics are natural antioxidants and are abundantly found in vegetables, fruits, seeds, pulp, peel, kernel, and oil [15].

Apricot retains tremendous nutrition, and its composition reveals that apricot fruit is a good source of minerals (iron, potassium, calcium, magnesium, sodium, phosphorus, copper, selenium, and zinc), vitamins A and C, fibers, thiamin, pantothenic acid, riboflavin, and niacin that are utilized for numerous purposes concerned with public health [16]. It has been reported that bitter apricot oil provides vitamins, fatty acids, and phenolic compounds, which play a vital role in antioxidant activity [17].

Essential oils extracted from fruits, herbs, and vegetables contain several beneficial biologically active compounds that exhibit potential against cancer, diarrhea, nausea, constipation, diabetes, kidney infections, nervous disorders, digestive disorders, inflammation, and cardiac disorders [7]. The antioxidants have been used as hepatoprotective, nervous system–defensive, nephroprotective, gastro-defensive, anti-inflammatory, and antiaging agents [18].

Phenolics, flavonoids, alkaloids, and tannins are among the substantial bioactive compounds present in apricot kernel, and it has been used as a folk medicine since ages [19]. Bitter apricot oil is industrially important and has an active elevation capacity like other essential oils without damaging the environment [20]. Oil derived from plant sources, such as essential oils, is considered environmentally friendly with no negative health implications on human health, resulting in its increasing demand across a variety of industries, including cosmetics, food, and pharmaceuticals [21]. The bitter apricot seed oil is extensively used for the treatment of skin health and skin infection [22] as a folk medicine. There is little work done on the exploration of bioactive component presence and evaluation of their medicinal and application potential in different modern industrial applications. The current study was therefore designed to evaluate the functional and therapeutic potential of the local bitter apricot seed oil along with the determination of nutritional and bioactive components present in it.

## 2. Material and Methods

### 2.1. Sample Collection

The seeds of the bitter apricot wild variety were obtained from Swat, Khyber Pakhtunkhwa province of Pakistan. The kernels were collected after the removal of the hard shells of seeds. The collected kernels were dried in a hot air oven at 40°C for 48 h. The plant and seed were identified by taxonomist Dr. Zahid Ullah, Associate Professor, Centre for Plant Science and Biodiversity, University of Swat. The voucher specimen of the plant was submitted to the herbarium of the Center for Plant Sciences and Biodiversity, University of Swat, “Swat,” Catalogue Number SWAT001380. Samples of the seed were also kept in the Food Microbiology and Bioprocess Technology Laboratory, University of Balochistan, for record.

### 2.2. Oil Extraction

The Soxhlet apparatus was used to extract oil following Tesfaye and Tefera [23] with slight changes. Bitter apricot seeds were grounded into powder, and 50 g of the sample was loaded into the thimble and placed in the Soxhlet extractor. Ethanol was used as extraction solvent, and oil was extracted for 6 h. The ethanol was then separated from the oil for further use.

### 2.3. Qualitative Analysis of Phytochemicals

The qualitative analyses for phytochemicals, that is, flavonoids, alkaloids, tannins, phlobatannins, saponins, terpenoids, steroids, cardiac glycosides, coumarins, quinones, and anthraquinones, were carried out via standard methods [24].

### 2.4. Determination of Total Phenolic Content (TPC) and Total Flavonoid Content (TFC)

TPC and TFC of BASKO were determined by standard colorimetric methods [25]. For TPC, gallic acid was used as a reference standard, and results were expressed as mg of gallic acid equivalent (GAE) per gram of the sample. Similarly, quercetin was used as a reference standard for the estimation of TFC, and results were expressed as milligrams of quercetin equivalent (QE) per gram.

### 2.5. Antioxidant Activity

Free radical scavenging activity was assessed using the 2,2,diphenyl-1-picrylhydrazyl (DPPH) assay [26]. Ethanol was used to prepare the DPPH reagent solution. DPPH (2 mL) solution was mixed with 0.5 mL BASKO and was kept at room temperature for 30 min. DPPH solution was used as a control, and vitamin C was used as a reference standard. The absorbance was recorded at 517 nm after the incubation. The test was conducted in triplicates, and the scavenging activity was calculated by the formula below:

 $$\begin{array}{c} \text{Scavenging percentage}=\left[\frac{\left(A_{0}-A_{1}\right)}{A0}\right]\times 100 \end{array}$$

Note: *A*_0_ is the control absorbance and *A*_1_ is the sample absorbance.

The IC_50_ values were determined by using different concentrations of oil for scavenging 50% of the DPPH free radical activities. The relationship curve was made by plotting the scavenging activities against different concentrations of oil and expressed in micrograms per milliliter.

### 2.6. Fourier Transform Infrared Spectroscopy

The chemical fingerprinting of BASKO was determined by FTIR spectrometer by recording 32 scans at 4 cm^−1^ resolution (Nicolet, Avatar 360). The samples' spectra were adjusted at 250–4500 cm^−1^ range.

### 2.7. Gas Chromatography and Mass Spectrometry

The detailed chemical profile of BASKO was analyzed by GC-MS (Model Perkin Elmer Clarus 500, United States) fitted with a VF-5 MS merged silica capillary column (30 m 0.25 mm, film thickness 0.25 mm) [27]. The BASKO was dissolved in 70% n-hexane (1:10, *w*/*v*) and subjected to GC-MS analysis to identify the chemical components. The temperature of the injector was planned to rise from 60° for 5 min to 230° for 45 min, with the injector temperature set at 230°. The initial and completion times were 2:50 and 72:00 min, respectively. Peak area equalization percentage was used to express the relative percentage of the chemical components in BASKO.

### 2.8. Antibacterial Activity

The Mueller–Hinton agar (MHA) was prepared and sterilized by autoclaving. The MHA was poured into sterile petri plates and left for solidification. Lawn of the target bacterial cultures, namely, *Escherichia coli*, *Salmonella typhi*, *Staphylococcus aureus*, *Klebsiella pneumoniae*, and *Pseudomonas aeruginosa* (adjusted to 1 × 10^8^ CFU/mL by comparing with 0.5 McFarland), was made over the surface of the MHA plates using sterile swabs. A 6-mm cork borer was used to create wells in the agar plates, and 250 *μ*L BASKO was pipetted into each well of the plates separately. The test was conducted along with positive and negative controls, doxycycline (an antimicrobial drug), and uninoculated media, respectively. The inoculated petri plates were incubated at 37°C for 24 h. After incubation, results were interpreted by evaluating the diameter of inhibition zones around the well [28].

### 2.9. Minimum Inhibitory and Minimum Bactericidal Concentration (MIC and MBC)

A twofold dilution procedure was used to analyze the MIC and MBC of BASKO in the Mueller–Hinton broth for both gram-negative and gram-positive bacterial species [29]. The dilution ratio of oil in the test medium was 1000, 500, 250, 125, and 62.5 *μ*L/mL. The 96-well plate was filled with 100 *μ*L of culture broth containing various dilutions along with controls. The lowest concentration that stopped the bacterial growth after 24–48 h at 37°C was considered MIC. The broth from the well with different concentrations was subcultured for bacterial growth using the standard plate count (SPC) procedure using freshly prepared nutrient agar plates and incubated at 37°C for 24 h. The appearance of no visible growth of concertation on the culture media plate after incubation was considered MBC. The experiments were run in triplicates of each experiment.

### 2.10. Antifungal Activity

The antifungal activity of the oil was determined by mixing 2 mL of BASKO with 1 mL of DMSO, followed by addition into a flask containing 24 mL of presterilized Sabouraud Dextrose Agar and poured onto the sterile petri plate. Then, 6-mm-diameter wells were carefully carved into solidified agar plates using cork borer and were filled with targeted fungal inoculum [30]. Fluconazole was used as a reference drug, while media free from oil and inoculated with target fungal species was employed as the positive control. The antifungal potency of BASKO was observed against *Mucor mucedo*, *Aspergillus niger*, *Aspergillus flavus*, *Epidermophyton floccosum*, and *Trichophyton tonsurans.* The calculation of growth inhibition was measured against the growth of target fungi in the control plates after 72 h of incubation at 35°C via the following equation. Fluconazole was used as a standard.

 $$\begin{array}{c} \text{Calculation}\%\text{inhibition of fungal growth}:\%\text{Inhibition}=100-\frac{\text{linear growth in test }\left(\text{mm}\right)\left(100\right)}{\text{linear growth in control }\left(\text{mm}\right)} \end{array}$$

### 2.11. Antileishmanial Assay (In Vitro)

The promastigotes of *Leishmania major* were taken from the previously isolated and preserved culture in the Food Microbiology and Bioprocess Laboratory, Department of Microbiology, University of Balochistan, Quetta. Shaheen et al. [11] were followed with slight modification to determine the antileishmanial potential of oil. The preserved leishmanial promastigotes were refreshed in a fresh culture medium. The NNN (Novy–Mac–Neal–Nicolle) biphasic medium with penicillin and streptomycin was used in the study for leishmanial growth. Test tubes were filled with 500 *μ*L of NNN media, whereas BASKO (1000 *μ*L) was poured into the first test tube, and subsequently twofold diluted serially (500, 250, 125, 62.5, and 31.2 *μ*L) and labelled accordingly. Thereafter, 20 *μ*L refreshed organism and 50 *μ*L of each dilution were added in a 96-well plate and incubated at 37°C for 24 h, and the same procedure was applied for standard. Glucantime was used as a standard drug, media without inoculum was negative, and media free from oil and inoculated with the organisms were used as positive controls. After incubation, 20 *μ*L nitro blue tetrazolium (NBT) salt stock solution was pipetted in a 96-well plate and incubated further at 37°C for 2 h. Absorbance was recorded at 630 nm in an ELISA reader, and results were interpreted via the following equation:

$$\begin{array}{cc} \; & \text{Cell viability}\%=\frac{A_{630}\text{ of test sample}}{A_{630}\text{ of control}}\times 100\\ \text{Inhibition}\%=100-\%\text{viability} \end{array}$$

### 2.12. Statistical Analysis

All experiments were conducted in triplicates, and the results are presented as mean values with standard deviation (±SD) from three replicates. To identify significant group differences (*p* < 0.05), one-way analysis of variance (ANOVA) and Tukey's tests were performed utilizing the SPSS statistical software package (SPSS Version 23.0). For IC_50_ estimation, data analysis was carried out using GraphPad Prism Version 9 (San Diego, United States).

## 3. Results

### 3.1. Phytochemicals

Phytochemicals are beneficial compounds with functional potential, and standard techniques were used to analyze the presence of phytochemicals in BASKO. The application of medicinal compounds derived from natural sources has significantly increased in health and pharmaceutical industries [14]. The finding of the current study demonstrated the presence of different important phytochemicals, namely, cardiac glycoside, coumarins, steroids, alkaloids, flavonoids, quinones, and terpenoids in BASKO as described in Table 1.

### 3.2. TPC and TFC

The phenolic and flavonoid compounds possess great medicinal importance and are considered good for human and animal consumption. The amount of TPC and TFC in the oil were calculated as 10.6 ± 1.32 mg GAE/g and 4.75 ± 0.11 mg QE/g, respectively.

### 3.3. DPPH Inhibition Assay

**Figure 1:**
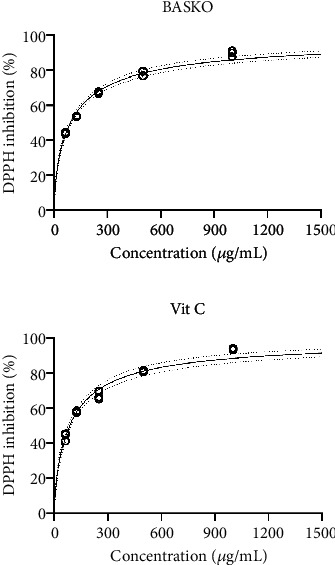
DPPH inhibition (percentage) by bitter apricot seed kernel oil.

It has been noted that DPPH radical scavenging activity of BASKO increased with the increase in oil concentration (Figure 1) and at the highest test concentration (1000 *μ*g/mL), 89.5% DPPH inhibition was observed. The IC_50_ value indicating the concentration of sample required 50% DPPH inhibition was calculated by the nonlinear regression curve fitting method and was observed as 90.44 *μ*g/mL (83.47–96.67 *μ*g/mL with 95% CI) [31]. The pattern of the dose versus response curve was predicted by extrapolation of data points beyond the experimental values by using the dose versus response variable slope, curve fitting method.

### 3.4. Fourier Transform Infrared Spectroscopy

The FTIR technique was used to detect the functional groups present in BASKO. The classification of functional groups was facilitated by the stretching, rocking, and bending of FTIR peaks (Table 2). The peaks were interpreted into functional groups in accordance with [32]. The C-H stretching indicated methylene's asymmetry at peaks 2973–2833 cm^−1^, whereas the aromatic rings, alkyl carbonate, and ester bend were determined at peaks 1750–1725 cm^−1^. The multiple compounds (aromatic ring, methyl asymmetric, carbonate ion, and methylene) were indicated at peaks 1490–1410 cm^−1^. Some compounds, including organic sulfate, aliphatic nitrocarbon, methyl, and nitrite ion, were demonstrated via bending and stretching vibration at peaks 399–1310 cm^−1^. The stretch of peaks 1240–1190 cm^−1^ was designed to present aromatic phosphate and ether. The peaks at 1190–1080 cm^−1^ stretching were designed to present sulfonate, secondary, tertiary, and tetra amine. Furthermore, the high variation of signals stretches and bends was observed at peaks 1150–1000 cm^−1^, indicating aliphatic fluoro compound, aromatic hydrocarbon, phosphate ion, organic siloxane, silicate ion, cyclic and alkyl-substituted ether, sulfate ion, and silicon. The stretching of peaks 1055–1000 cm^−1^ demonstrated the presence of aliphatic fluoro compound, aliphatic phosphate, cyclohexane ring, silicon, primary amine, and silicate ion. The 800–700 cm^−1^ peaks were designed to indicate aromatic hydrocarbons, aliphatic chloro groups, and methylene, whereas 500–430 cm^−1^ peaks described the aryl and polysulfides.

### 3.5. Gas Chromatography–Mass Spectrometry

GC-MS analysis revealed that different fatty acids are present in BASKO, including omega-6 and omega-9, oleic acid (monounsaturated fatty acid), and palmitic acid (saturated fatty acid). Apart from these, BASKO contained the following fatty acids: stearic acid (saturated fatty acid), margaric acid (crystalline saturated fatty acid), caprylic acid (saturated fatty acid), adipic acid (medium chain fatty acid), butanoic acid (saturated short chain fatty acid), caproic acid (saturated fatty acid), and valeric acid (straight chain fatty acid). In addition to fatty acids, numerous other beneficial compounds were found in it, such as aromatic compounds, benzene derivatives, phenols, ethyl and methyl derivatives, aldehydes, naphthalene products, nicotinic acid (vitamin B), pyrrole, ketones, alcohol, silicon derivatives, and sulfides. Furthermore, several acids comprising salicylic acid, benzoic acid, cinnamic acid, butanoic acid, propanoic acid, fumaric acid, malonic acid, acetic acid, hexynoic acid, and numerous other compounds are found in BASKO as present in Table 3.

### 3.6. Antibacterial Activity

The BASKO exhibited good antibacterial activity against *S. aureus* (25 ± 1.01 mm), *E. coli* (20.3 ± 2.08 mm), and *S. typhi* (19.3 ± 2.51 mm) compared to *P. aeruginosa* (17 ± 2 mm) and *K. pneumoniae* (16.6 ± 1.52 mm). The results show good potency of the oil against target bacterial species (Table 4).

### 3.7. MIC and MBC

In the present study, the BASKO was found to be highly potent against target bacterial species. The MIC value recorded was 250 *μ*L/mL for *K. pneumoniae*, *S. typhi*, *P. aeruginosa*, and *S. aureus*, whereas 62.5 *μ*L/mL for *E. coli*. The MBC of BASKO was noted at the concentration of 62.5 *μ*L/mL on *E. coli* and at the concentration of 250 *μ*L/mL on *S. aureus* and *S. typhi*, while it inhibited the growth of *K. pneumoniae* and *P. aeruginosa* at 250 *μ*L/mL and exhibited bacteriostatic effect (Table 5).

### 3.8. Antifungal Activity

The *E. floccosum*, *M. mucedo*, *A. niger*, *T. tonsurans*, and *A. flavus* were used as target test organisms to determine the antifungal activity of the oil by measuring the growth inhibition percentage. BASKO was effective against all the fungal species, and the inhibition percentages of 90 ± 3%, 77.3 ± 2.08%, 69.6 ± 3.51%, 74.3 ± 2.56%, and 78.3 ± 2.51% were observed against *A. flavus*, *T. tonsurans*, *E. floccosum*, *A. niger*, and *M. mucedo*, respectively (Table 6). The antifungal activity of the standard drug fluconazole was slightly higher than the oil.

### 3.9. Antileishmanial Activity

**Figure 2:**
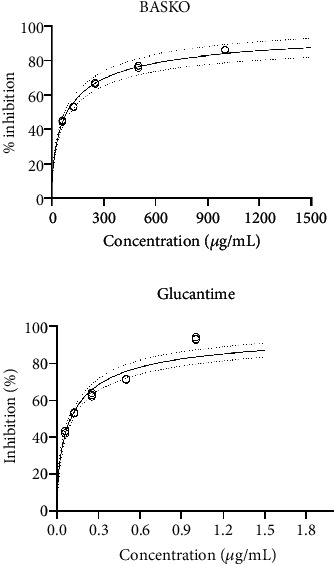
Antileishmanial activity of bitter apricot seed kernel oil against *L. major*.

It was found in the study that *L. major* is sensitive to BASKO with the IC_50_ = 89.75 *μ*g/mL with 95% confidence interval (83.72–95.11 *μ*g/mL), whereas the standard drug glucantime had IC_50_ = 0.099 *μ*g/mL with 95% confidence interval (0.085–0.11 *μ*g/mL) (Figure 2).

## 4. Discussion

A qualitative examination of phytochemicals is required to identify bioactive constituents in plants and plant products. Due to metabolic processes occurring at various stages of a plant's growth, phytochemicals such as tannins, terpenoids, glycosides, and quinones are produced. These products, such as coumarins, alkaloids, terpenoids, flavonoids, and phenolics, were detected in our study, and some of these biocomponents have been reported by Shaheen et al. [11] in apricot leaf extract and oil [33]. It has been reported that antioxidant and antibacterial potential can be directly associated with the presence of alkaloids, phenolics, and flavonoids [34].

The *Prunus* species are abundant in flavonoids, phenolics, and other bioactive components. Kasapoğlu, Kahraman, and Tornuk [35] reported the TFC and TPC 1.015 mg CE/g and 1.206 mg GAE/g in the extract of apricot pomace, whereas Joujou et al. [34] reported the presence of flavonoids and phenolic components in different seeds, including bitter apricot, with varying concentrations. These results are in agreement with our study of bioactive constituents. We got slightly higher TPC components (10.6 ± 1.32 mg GAE/g) compared to the earlier testified (9.8 GAE/g) in a similar study [36]. The TFC amount reported by Juhaimi et al. [37] was higher in roasted apricot kernel (17.33 mg CE/g), sweet apricot (0.468 mg QUE/g), and bitter apricot (8.099 mg QUE/g) compared to our results.

The radical scavenging activity of the BASKO was 64%, lower (79.85%) than that reported by Kasapoğlu, Kahraman, and Tornuk [35] and higher (44%) than that reported by Cheaib et al. [38] in apricot pomace. In some other studies, the antioxidant activity of sweet apricot and bitter apricot seed extracts has been reported as 87.7% and 20%, respectively [39]. Different Pakistani apricot cultivars have been analyzed for their radical scavenging activity and were found in the range between 55% and 83% [40] and 45% and 90%. [3].

The FTIR illustrations of bands and stretches are used to investigate the existence of chemical bonds. Numerous chemicals, including hydrocarbons, aromatic, aliphatic, and cyclic groups, were detected by the FTIR technique in BASKO. The current findings are in agreement with [41], which evidently described similar results in peach. Researches indicate that the apricot kernel contains the following chemical groups: C=O, CH3, OH, C-O, CH, and C-O-H [33]. Recent research by [42] evaluated the bending and rocking of CH, ether vibrations, and aromatic ring vibrations at peaks of 756–569, 1196–1088, and 1742 cm^−1^, correspondingly. Aliphatic nitrile, C=C stretch, N-H stretch, P-CL stretch, C-C stretch, methylene, and secondary amide were described previously [43] and were attributed to several peaks in the apricot kernel.

According to the literature, apricot oil contains a lot of fatty acids, aldehydes, hydrocarbons, ketones, phenols, alcohol, and aromatic chemicals. The current outcomes are in accordance with [44], which states the occurrence of organic acids, fatty acids, aldehydes, benzene rings, aromatic compounds, alcohol, esters, phenols, and hydrocarbons in the bitter apricot kernel. The fatty acids' presence is in accordance with [33, 45–48] stated butanoic acid (saturated short-chain fatty acid), stearic acid (saturated fatty acid), palmitic acid (monosaturated fatty acid omega-6), and oleic acid (monosaturated fatty acid omega-9) in apricot. Other fatty acids such as caproic acid (saturated fatty acid), valeric acid (straight chain fatty acid), stearic acid, caprylic acid (saturated fatty acid), palmitic acid, adipic acid (medium chain fatty acid), and oleic acid were reported by Kumar et al. [49] in chaff flower. The margaric acid (crystalline saturated fatty acid) was reported in a recent study by Moghadasian et al. [50] in Iranian caper. It has been reported that apricots contain fumaric acid, quinolinecarboxylic acid, glutaric acid, silicon derivatives, succinic acid, and derivatives of acetic acid [44]. The apricot extract contains a variety of chemicals, including furan, furazano, alcohol, phosphates, ethers, amine groups, ketones, cyclic and acyclic compounds, methyl and ethyl derivatives, and numerous compounds with the benzene ring, according to an earlier study [51]. Reduced risk of cancer, Alzheimer's disease, birth abnormalities, and homocysteine levels are all advantages of folic acid for the heart and brain [52]. Earlier, Prasad and Rao [53] reported the occurrence of sebacic acid in castor oil, which has applications in cosmetics, aromatherapy, candles, and painting products. The manifestation of several beneficial compounds, including benzoic acid, propanoic acid, propionic acid, fumaric acid, carboxylic acid, malonic acid, hexynoic acid, butanoic acid, furanone, acetamide, phenol, cyclohexane, several diones, pyrrole, sulfides, benzene derivatives, ethyl derivatives, and methyl products, has reportedly been found in olive oil waste [54]. Recently, it was stated [55] that salicylic acid, cinnamic acid, and carbamate, together with their derivatives, are natural insecticides. Niacin, also known as nicotinic acid, is a type of vitamin B complex that helps with heart and neurological system disorders, diabetes, and skin infections [56]. The oil gains more prominence in the food, cosmetic, and medicine industries due to the presence of organic acids, fatty acids, methyl and ethyl compounds, natural preservatives, natural insecticides, antiaging compounds, and vitamins [2]. Naturally occurring preservatives include benzoic acid, ascorbic acid, cinnamic acid, sulfites, benzoates, phosphates, and propionates [21]. The EOs naturally contain a wide range of beneficial compounds, such as fatty acids, hydrocarbon derivatives, ketones, benzene derivatives, alcohols, organic acids, oxides, aldehydes, phenols, and esters, and such compounds are extensively used in cosmetics because they comprise no side effects; have a pleasant aroma, antioxidant activity, and skin elasticity; have antimicrobial and antiaging properties; and therefore can treat scars, acne, and stretch marks. The EOs have gained a dramatic increase and become prominent, particularly in the perfume and cosmetic industries [57].

The apricot and its seed are an inexpensive source of numerous substances that are effective against bacteria that are resistant to multiple drugs [22]. We found that BASKO has remarkable activity against a wide range of gram-positive and gram-negative bacteria; however, *S. aureus* and *S. typhi* were among the most susceptible to BASKO, with inhibition zones ranging 25 ± 1.01 and 19.3 ± 2.51 mm, which are in agreement with other studies [58]. The present analysis is relevant to the study of Lee et al. [59]; they reported complete growth inhibition of *Salmonella typhimurium*, *S. aureus*, *E. coli*, *P. aeruginosa*, and *S. typhi* by apricot essential oil. Previously, Gupta et al. [60] stated that apricot was found exceptionally effective against *Helicobacter pylori* and *Mycobacterium tuberculosis*, respectively.

The current analysis is in agreement with [61], and they reported similar concentrations for *S. aureus* and *E. coli*. The MIC and MBC analyses are in agreement with Abtahi, Ghazavi, and Karimi [62], and they stated that bitter apricot extract was found most effective against *S. aureus.* Earlier, Rashid et al. [58] reported that a methanolic extract of apricot was active against various gram-negative (*Enterobacter aerogenes*, *P. aeruginosa*, *Shigella dysenteriae*, *E. coli*, *S. typhi*, *K. pneumonia*) and gram-positive (*Streptococcus pyogenes*, *Streptococcus faecalis*, *S. aureus*, *Micrococcus luteus*, and *Corynebacterium diphtheriae*).

This study revealed that BASKO is potent against fungal isolates. BASKO showed fungal inhibition of *A. flavus* (90 ± 3%), *T. tonsurans* (77.3 ± 2.08%), and *E. floccosum* (76.6 ± 3.51%). Our study outcomes resemble the study of Hashemi and Raeisi [63], and they demonstrated that apricot gum was active against a variety of fungi, including *Alternaria alternata*, *Fusarium oxysporum*, and *A. flavus*. Apricot oils have been found effective against *Malassezia furfur* and *Candida albicans* [59, 64]. Apricots include a number of bioactive substances that enhance their potency against antifungal effects [65].

The antileishmanial activity of this study is in agreement with Shaheen et al. [11], and they reported apricot leaf extract as a potent antileishmanial agent. Minaiyan et al. [66] reported apricot usage in the treatment of numerous protozoal infections. The apricot contains a number of phytochemicals (saponins, terpenoids, tannins, flavonoids, phenols, quinine, coumarins, and alkaloids) that are responsible for its medicinal properties. The antiparasitic activity of coumarins, flavonoids, phenolics, and alkaloids has been reported [7].

## 5. Conclusion

The present study concludes that bitter apricot kernel oil is an important source of biologically important components. It contains useful chemical compounds that possess several beneficial potentials, such as antimicrobial activities against a wide range of bacteria and fungi, antiparasitic, antioxidant, and antispoilage activity. This study revealed that this oil has the potential to be used as a disinfectant, insecticidal, preservative, cosmetic ingredient, and antimicrobial agent. The oil shows good potency against a variety of fungi used in this study, thus confirming it as a good food preservative agent.

## Figures and Tables

**Table 1:** Qualitative analysis results of phytochemicals present in bitter apricot seed kernel oil.

**S#**	**Compounds tested**	**Result**
1	Steroid	Present
2	Saponins	Absent
3	Quinones	Present
4	Phlobatannins	Absent
5	Coumarins	Present
6	Flavonoids	Present
7	Terpenoids	Present
8	Anthraquinones	Absent
9	Alkaloids	Present
10	Cardiac glycoside	Present
11	Tannins	Absent

**Table 2:** Peaks results of the FTIR spectra for bitter apricot seed kernel oil.

**Wavenumber (cm^−1^)**	**Main attribution**	**Link type**	**Vibration mode**
447.75	Aryl sulfide	S-S	Stretch

478.31	Poly sulfide	S-S	Stretch
	Aryl sulfide	S-S	Stretch

721.33	Aliphatic chloro compound	C-Cl	Stretch
	Aromatic hydrocarbon	C-H (out of plane)	Bend
	Methylene	(CH_2_)_n_	Rocking

1049.2	Primary amine	CN	Stretch
	Aliphatic phosphate	P-O-P	Stretch
	Silicon	Si-O-Si	
	Silicate ion		
	Cyclohexane ring		
	Aliphatic fluoro compounds	C-F	Stretch

1091.63	Organic siloxane	Si-O-Si	
	Silicon		
	Silicate ion		
	Sulfate ion		
	Phosphate ion		
	Aromatic hydrocarbon	C-H (in plane)	Bend
	Aliphatic fluoro compound	C-F	Stretch
	Cyclic ether large ring	C-O	Stretch
	Alkyl-substituted ether	C-O	Stretch

1161.07	Sulfonate		
	Tetra amine	CN	Stretch
	Cyanate	C-OCN	Stretch
	Secondary amine	CN	Stretch
	Tertiary amine	CN	Stretch

1234.36	Aromatic ether	Aryl-O	Stretch
	Aromatic phosphate	P-O-C	Stretch

1377.08	Methyl	C-H asym	Stretch
	Organic sulfate		
	Nitrate ion		
	Aliphatic nitro carbon		

1458.08	Carbonate ion		
	Aromatic ring	C=C-C	Stretch
	Methylene	C-H	Bend
	Methyl asym	C-H	Bend

1743.53	Aromatic ring		Bend
	Ester		
	Alkyl carbonate		

2854.45	Methylene asym	C-H	Stretch

2923.88	Methylene	C-H asym	Stretch

**Table 3:** Chemical profile of the bitter apricot seed kernel oil determined by GC-MS.

**Compound**	**Retention time (min)**	**Area**	**Area (%)**	**Mol. formula**	**Mol. weight**
Pentanoic acid (valeric acid)	4.216	17,076	0.52	C_9_H_16_N_2_O_2_	184
4,11-Dihexyl-16,16-dimethyl-1,14-dioxa-4,11-diazacycloheptadecane-3,12-dione	4.785	22,517	0.68	C_27_H_52_N_2_O_4_	468
DL-Homocystine (sulfide)	4.900	20,794	0.63	C_8_H_16_N_2_O_4_S_2_	268
Azocin	5.310	20,933	0.64	C_7_H_15_N	113
Piperazine, 1-acetyl-4-(2-methyl-4-nitro)	8.015	27,637	1.02	C_13_H_17_N_3_O_3_	263
3-Isopropyl imidazolidine-2,4-dione	8.313	11,360	0.35	C_6_H_10_N_2_O_2_	142
Octanoic acid (caprylic acid)	10.215	7278	0.70	C_10_H_18_Cl_2_O_3_	256
L-Serine, dihydrogen phosphate	14.295	20,669	0.63	C_3_H_8_NO_6_P	185
Benzoic acid	15.060	44,430	1.35	C_10_H_12_N_2_O_2_	192
Phenol, 2,6-dichloro-4-nitro	27.945	5269	0.16	C_6_H_3_Cl_2_NO_3_	207
4,4⁣′-(Hydroxymethylene)diphthalic anhydride	28.381	6165	0.19	C_17_H_8_O_7_	324
Acetophenone, 4⁣′,4⁣^″′^-ethylenedi	29.820	12,833	0.39	C_18_H_18_O_2_	266
2-(Bromomethyl)benzyl alcohol, TMS derivative	30.175	21,634	0.66	C_11_H_17_BrOSi	272
2-Chloro-2,5-dimethyl-5-propyl-2,5-disilaoctane	31.045	18,107	0.55	C_11_H_27_ClSi_2_	250
[1,2,5]Oxadiazolo[3,4-b]pyrazine	31.250	5651	0.17	C_4_Cl_2_N_4_O	190
Tetraethyl 4,4⁣′-(1,3-phenylene)bis(1,4-dihydro-2,6-dimethyl-3,5-pyridinedicarboxylate)	31.305	17,340	0.98	C_32_H_40_N_2_O_8_	580
1-Propanone, 1-(2,6-dimethyl-4-propoxyphenyl)-3-(1-piperidyl)	32.777	18,277	0.56	C_19_H_29_NO_2_	303
Silicic acid	48.620	9383	0.29	C_10_H_28_O_4_Si_3_	296
Carbamic acid (insecticide)	33.371	14,374	0.44	C_20_H_23_N_3_O_3_	353
Methyl 3-hydroxybenzoate, TMS derivative	33.576	40,950	1.25	C_11_H_16_O_3_Si	224
Acetic acid	34.105	11,563	0.35	C_6_H_12_O_2_S	148
Beta.-D-galactopyranoside, methyl 2,3-bis-O-(trimethylsilyl)-, cyclic butylboronate	35.613	32,253	0.98	C_17_H_37_BO_6_Si_2_	404
Carboxylic acid	35.773	20,712	0.63	C_12_H_18_O_2_	194
(3,4-Dihydroxyphenyl)hexylamine	36.110	14,231	0.43	C_12_H_19_NO_2_	209
Phenylephrine, bis(trimethylsilyl) ether (nasal relieve)	52.769	7522	0.23	C_15_H_29_NO_2_Si_2_	311
Tris(tert-butyldimethylsilyloxy)arsane	37.740	15,691	0.48	C_18_H_45_AsO_3_Si_3_	468
Butyric acid, methyl ester	39.638	9806	0.30	C_7_H_13_NO_4_	175
9-Selena-10-cobaltabicyclo[6.3.0]undeca-1(8),2-diene, 11-methyl-, pentamethylcyclopentadienyl	39.679	6038	0.18	C_20_H_29_CoSe	408
Ethyl homovanillate, TMS derivative	57.298	24,172	0.74	C_14_H_22_O_4_Si	282
2,4(1H,3H)-Pyrimidinedione, 6-chloro-5-nitro	40.960	9762	0.30	C_4_H_2_ClN_3_O_4_	191
Methyl benzoate	41.030	8078	0.93	C_16_H_24_O_4_Si	308
3-[4-Chloro-.alpha.,.alpha.,.alpha.-trifluoro-m-tolyl]-5-methylrhodanine	43.330	45,504	1.38	C_11_H_7_ClF_3_NOS_2_	325
Methyl mandelate, TMS derivative	43.490	12,631	0.38	C_12_H_18_O_3_Si	238
5-Hydroxymethyluracil, 3TMS derivative	44.310	13,757	0.42	C_14_H_30_N_2_O_3_Si_3_	358
Silanamine	44.574	8280	0.25	C_14_H_27_NOSi_2_	281
Hexasiloxane, tetradecamethyl	44.650	16,109	0.49	C_14_H_42_O_5_S_i6_	458
Silane, methylvinyl(phenoxy)ethoxy	44.742	5689	0.17	C_11_H_16_O_2_Si	208
Nicotinic acid (niacin)	45.024	26,948	1.30	C_9_H_10_N_2_O_4_	210
Butanesulfinamide, TMS derivative	47.367	7022	0.21	C_7_H_10_F_9_NOSSi	355
Benzene	48.019	29,848	0.91	C_12_H_22_Si_2_	222
Oleic acid	49.832	14,246	0.43	C_25_H_39_Cl_2_NO_2_	455
o-Anisaldehyde, semicarbazone	52.815	21,334	0.65	C_9_H_11_N_3_O_2_	193
nor-Mephedrone	53.015	6836	0.21	C_10_H_13_NO	163
2-[4-(1,2-Diphenyl-but-1-enyl)-phenoxy]-ethylamine	53.220	53,060	1.61	C_24_H_25_NO	343
2-Propenamide, N-(1-cyclohexylethyl)	53.325	17,862	0.54	C_11_H_19_NO	181
(E)-Prop-1-en-1-yl propanedithioate	53.654	24,125	0.73	C_6_H_10_S_2_	146
4-Amino-5-methyl-2-trichloromethyloxazolidine	53.846	48,747	1.48	C_5_H_9_Cl_3_N_2_O	218
Myrtanol, 2-mercapto	54.018	9439	0.29	C_10_H_18_OS	186
1,2-Digermacyclopentane, 1,1,2,2-tetramethyl	54.383	15,924	0.48	C_7_H_18_Ge_2_	250
Methanol, TBDMS derivative	58.420	13,187	0.40	C_15_H_24_O_2_Si	264
Dimethylphenol, TMS derivative	59.900	38,042	1.16	C_11_H_18_OSi	194
4-Methyl-3-(3-nitrophenyl)-6-phenyl-5,6-dihydro-4H-[1,2,4,5]oxatriazine	61.467	9953	0.30	C_15_H_14_N_4_O_3_	298
*cis*-Cyclohexane-1,3-dicarboxamide	62.613	19,630	0.93	C_8_H_14_N_2_O_2_	170
5-[.alpha.-Methoxyethyl]tubercidin, hemihydrate	63.530	30,019	0.91	C_14_H_20_N_4_O_5_	324
Furazano	64.254	6568	0.20	C_8_H_8_N_4_O_4_	224
Glutaric acid	64.460	10,712	0.33	C_15_H_23_F_3_O_4_	324
Methylzinc propoxide	65.240	7325	0.22	C_4_H_10_OZn	138
Succinic acid	66.699	25,861	0.79	C_12_H_22_O_5_	246
1H-Benzoimidazole, 2-benzyl-1-isobutyl	66.362	13,901	0.42	C_18_H_20_N_2_	264
Acetamide (stablizer, pasticizer)	66.791	17,955	0.55	C_21_H_25_N_3_O_2_S	383
4-t-Butyl-6-dimethylaminomethyl-2-[4-dimethylaminophenyl]phenol	67.042	21,157	0.64	C_21_H_30_N_2_O	326
1H-2,5-Benzoxazocine, 3,4,5,6-tetrahydro-1-phenyl	68.084	21,852	0.66	C_16_H_17_NO	239
Naphthalene,1-(1-cyclohexen-1-yl)	68.304	22,212	0.68	C_16_H_16_	208
2-Methylthiomethyl-4-(1-methylthioethyl)thietane	69.203	7909	0.24	C_8_H_16_S_3_	208
2-Methoxy-4,6-bis(pyrrolidin-1-yl)-1,3,5-triazine	69.925	18,843	0.57	C_12_H_19_N_5_O	249
5-(2H-1,3-Benzodioxol-5-yl)-3-(2-hydroxyphenyl)-4,5-dihydropyrazole-1-carbaldehyde	71.325	10,668	0.32	C_17_H_14_N_2_O_4_	310
Propionic acid (herbicides)	3.627	5892	0.92	C_4_H_4_F_3_N_3_O_3_	199
Hexynoic acid (flavoring)	5.570	14,723	0.24	C_6_H_8_O_2_	112
Phthalic acid (benzenedicarboxylic acid)	25.339	36,478	0.60	C_23_H_36_O_4_	376
Hexanedioic acid (adipic acid)	27.794	5794	0.43	C_10_H_16_Cl_2_O_4_	270
Quinolinecarboxylic acid	30.790	19,556	0.32	C_10_H_9_NO_4_	207
Octadecanoic acid (stearic acid)	35.882	22,200	0.36	C_22_H_44_O_4_Si	400
Butanoic acid	58.790	24,041	0.39	C_6_H_11_BrO_2_	194
Salicylic acid	55.760	16,408	0.50	C_18_H_26_N_2_O_3_Si_22_	374
Heptadecanoic acid (margaric acid)	61.300	17,664	0.29	C_17_H_34_O_2_	270
Propanoic acid	65.879	14,326	0.24	C_8_H_14_O_4_S_2_	238
Malonic acid (propanedioic acid)	69.857	30,821	0.91	C_23_H_28_O_6_	400
Decanedioic acid (sebacic acid)	70.021	31,742	0.95	C_12_H_22_O_4_	230
Furanone (perfumer)	10.118	3694	0.36	C_11_H_16_O_3_	196
Folic acid	41.685	2624	0.26	C_19_H_19_N_7_O_6_	441
Fumaric acid	9.518	3733	0.04	C14H24O4	256
Hexadecanoic acid (palmitic acid)	27.794	5794	0.69	CH_3_(CH_2_)_14_COOH	256
Cinnamic acid, (E)-, TMS derivative	33.900	4289	0.67	C_12_H_16_O_2_Si	220
Hexanoic acid (caproic acid)	32.462	12,042	0.37	C_6_H_12_O_2_	116

**Table 4:** Antibacterial activity of bitter apricot seed kernel oil.

**Organism**	**Zone of inhibition**	**Doxycycline**
*Escherichia coli*	20.3 ± 2.08^b^	23 ± 0.13
*Salmonella typhi*	19.3 ± 2.51^b^	17 ± 0.21
*Klebsiella pneumoniae*	16.6 ± 1.52^b^	22 ± 0.35
*Pseudomonas aeruginosa*	17 ± 2^b^	18 ± 1.67
*Staphylococcus aureu*s	25 ± 1.01^a^	14 ± 1.81

*Note:* Results are displayed as a zone of inhibition and standard deviation. Different superscript letters within the column indicate means which are significantly different (*p* < 0.05).

**Table 5:** Minimum inhibition and minimum bactericidal concentration of bitter apricot seed kernel oil.

**Organism**	**MIC (*μ*L/mL)**	**MBC (*μ*L/mL)**
*Escherichia coli*	62.5	62.5
*Salmonella typhi*	250	250
*Staphylococcus aureus*	250	250
*Klebsiella pneumoniae*	250	250
*Pseudomonas aeruginosa*	250	250

**Table 6:** Antifungal activity of bitter apricot seed kernel oil.

**Organisms**	**BASKO**	**Fluconazole**
	**Inhibition (%)**	
*Trichophyton tonsurans*	77.3 ± 2.08^bc^	79 ± 0.82
*Epidermophyton floccosum*	69.6 ± 3.51^c^	74 ± 1.63
*Aspergillus niger*	74.3 ± 2.56^bc^	97 ± 0.82
*Aspergillus flavus*	90 ± 3^a^	93 ± 2.45
*Mucor mucedo*	78.3 ± 2.51^b^	95 ± 0.41

*Note:* Results are presented as inhibition percentage and SD. Different superscript letters within the column indicate means which are significantly different (*p* < 0.05).

## Data Availability

A major portion of the data is included in this manuscript. Any additional data needed can be made available upon reasonable request.
